# Burden of depression and anxiety disorders per disease codes in patients with lymphoma in Germany

**DOI:** 10.1007/s00520-021-06677-w

**Published:** 2021-11-10

**Authors:** Marie-Kristin Tilch, Peter R. Galle, Jörn M. Schattenberg, Karel Kostev, Christian Labenz

**Affiliations:** 1 Department of Internal Medicine III, University Medical Center of the Johannes Gutenberg-University, Mainz, Germany; 2 Department of Internal Medicine I, University Medical Center of the Johannes Gutenberg-University, Langenbeckstrasse 1, 55131 Mainz, Germany; 3 Epidemiology, IQVIA, Frankfurt am Main, Germany

**Keywords:** Cancer, Anxiety disorders, Depression, Lymphoma, Hodgkin’s lymphoma, Oncology

## Abstract

**Purpose:**

The aim of this study was to explore the incidence of depression and anxiety disorder diagnoses in a large German cohort of patients with Hodgkin lymphoma (HL) and non-Hodgkin lymphoma (NHL) diagnoses in comparison to patients without cancer over a 10-year time frame.

**Methods:**

Patients with HL (*n*=687) and NHL (*n*=4130) were matched to cohorts without a cancer diagnosis (*n*=687 and 4130) by age, sex, and yearly consultation frequency. The primary outcome of the study was the incidence of depression and anxiety disorders. The relationship between lymphoma, separated into HL and NHL, and both depression and anxiety disorders was investigated using Cox regression models.

**Results:**

We compared 687 patients with HL with 687 matched non-cancer individuals and 4130 patients with NHL with 4130 matched non-cancer individuals. Within 10 years of the index date, 24.0% of patients with HL and 22.3% of patients with NHL were diagnosed with depression. Anxiety disorders were diagnosed in 6.7% and 5.3% of patients with HL and NHL, respectively. On regression analyses, HL (HR 2.30, 95% CI 1.65–3.21, *p*<0.001) and NHL (HR 2.09, 95% CI 1.81–2.41, *p*<0.001) were positively associated with incident depression. The HR for anxiety disorders was 1.64 (95% CI 1.24–2.16, *p*<0.001) in patients with NHL, while HL was not associated with incident anxiety disorders (HR 1.21, 95% CI 0.71–2.07, *p*<0.478).

**Conclusion:**

Lymphoma constitutes a risk factor for emerging depression and anxiety disorders. Following the diagnosis of lymphoma, screening and strategies to prevent the occurrence of these diseases seem warranted.

## Introduction

Depression and anxiety disorders (compromising panic disorder, agoraphobia, social phobia, generalized anxiety disorder, specific phobias, and separation anxiety disorder) are common mental health disorders in Germany [1]. In 2019, the prevalence of depressive symptoms was 9.2% according to the Robert-Koch-Institute [2]. The prevalence of anxiety disorders was even higher with a prevalence of about 15% [3].

In patients with cancer that suffer from cancer-related psychological distress, the risk of depression and anxiety disorders is even higher [4]. Unrecognized mental diseases have a strong impact on a patients’ quality of life and can lead to an increased mortality [5]. Furthermore, depression is associated with impaired socioeconomic outcomes due to prolonged sick leave, increased risk of disability pension, and difficulties in maintaining family life [6–8]. Therefore, patients following a cancer diagnosis should be screened for the occurrence of depression or anxiety disorders. The HL guidelines recommend that the individual need for psycho-oncological care should be addressed and patients should be offered the opportunity for a psycho-oncological consultation [9].

Generally, a higher age and female sex are both associated with higher lifetime risk of depression and anxiety disorders in the general population [10]. However, mental health status may differ in cancer survivors depending on the type of cancer and its outcome after treatment, e.g., patients with skin or prostate cancer have a lower incidence of depression as patients with gynecological or lung cancer [10]. There have only been a few longitudinal studies focusing on mental health in patients with cancer including lymphoma patients, e.g., the survey of Boyes et al. [11]. However, research on mental health in patients with hematological cancer is limited, especially with focus on malignant lymphoma.

Malignant lymphoma is generally distinguished into Hodgkin lymphoma (HL) and non-Hodgkin lymphoma (NHL). The diagnosis relies on clinical examination, and morphological and immunophenotypic assessment from a lymph node biopsy. HL is an uncommon B cell malignancy, accounting for about 10% of all lymphoma, with high incidence in young adults (median age at diagnosis: 39 years) [12]. Treatment optimization within the last decades results in remarkably high survival rates with an estimated 5‐year survival rate of 89.9% for those diagnosed between age 20 and 64 years [12]. NHL represents a vast spectrum of diseases, varying from indolent to aggressive B or Tcell malignancies with more than 50 different subtypes listed in the latest World Health Organization classification [13]. Among B-NHL, the most common aggressive lymphoma is diffuse large B cell lymphoma (DLBCL), which is curable in 60–70% of patients with chemoimmunotherapy. Follicular lymphoma (FL) is the most common indolent lymphoma, following a relapsing-remitting course and therefore considered incurable.

Although therapy regimens for malignant lymphoma have been improved over the last decades, a substantial proportion of patients does not experience cure or long-term disease control, which may negatively affect the occurrence of depression or anxiety disorders in these patients. However, data on the incidence of these diseases in patients with lymphomas are currently scarce. Therefore, we hypothesized that patients with lymphoma have a higher incidence of depression and anxiety disorders and explored the coded incidence in a large cohort of patients with HL and NHL in Germany.

## Methods

### Database

This study is a retrospective cohort study and was based on secondary data from the longitudinal Disease Analyzer database (IQVIA), which compiles drug prescriptions, diagnoses, and basic medical and demographic data of outpatients obtained directly and in anonymous format from computer systems used in the practices of general practitioners [14–16]. Diagnoses (International Classification of Diseases, 10^th^ revision [ICD-10]), prescriptions (European Pharmaceutical Market Research Association [EPhMRA] Anatomical Therapeutic Chemical Classification [17] system), and the quality of reported data are monitored by IQVIA based on several criteria (e.g., completeness of documentation and linkage between diagnoses and prescriptions). The data are generated directly from the computers in the physicians’ practices via standardized interfaces and provide daily routine information on patients’ diseases and therapies. IQVIA uses summary statistics from all physicians in Germany published yearly by the German Medical Association to determine the panel design for the Disease Analyzer database according to the following strata: specialist group, German federal state, community size category, and age of the physician. Rathmann et al. could show a good agreement between the incidence or prevalence of major chronic diseases (for example, cancer, dementia, diabetes) in the Disease Analyzer database and German reference data [15].

### Study population

**Fig. 1:**
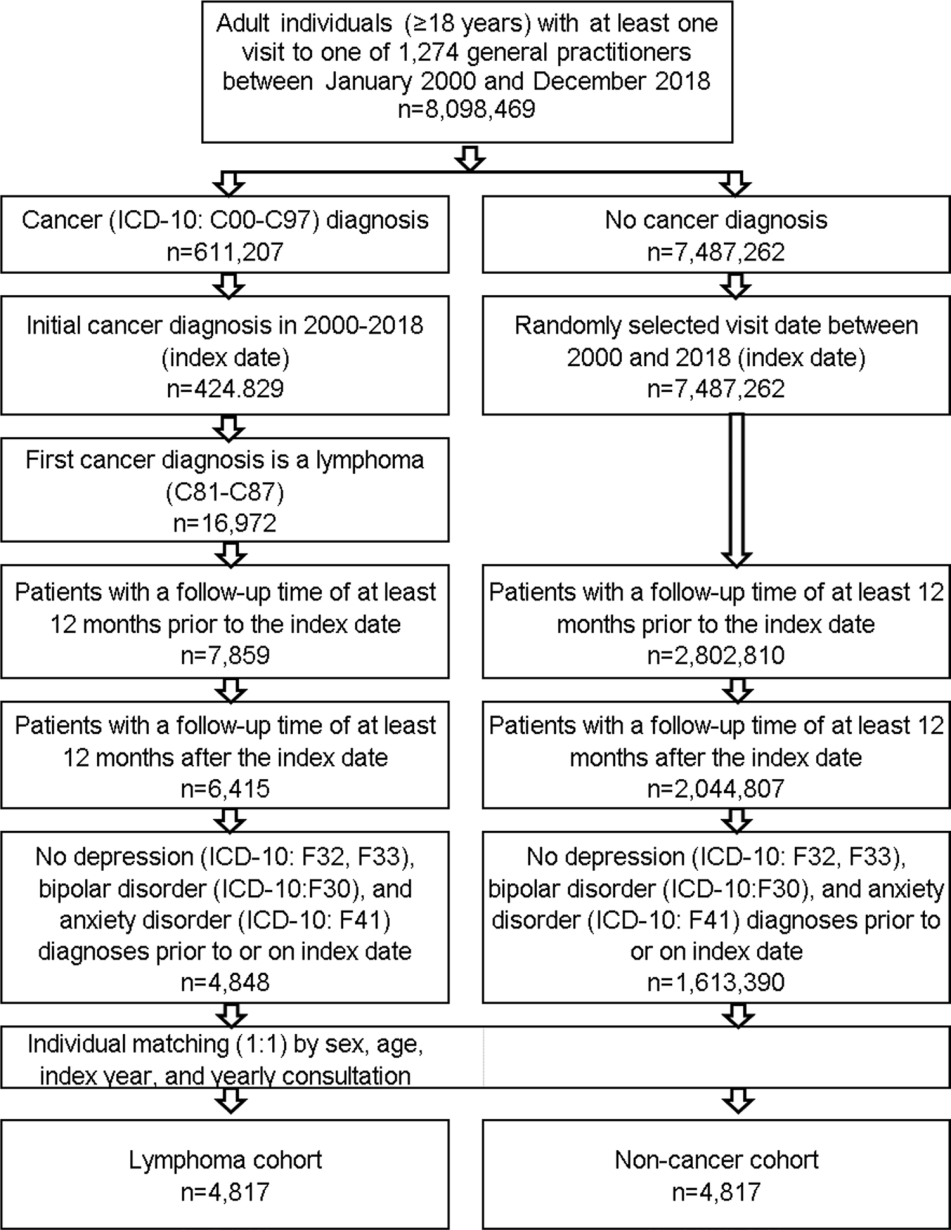
Selection of study patients

This study included patients who were coded for the first time with a diagnosis of Hodgkin (ICD-10: C81) or non-Hodgkin (ICD-10: C82-C88) lymphomas in one of 1274 general practices in Germany between January 2000 and December 2018 (index date). Included patients were aged ≥18 years at the index date and had never been diagnosed with cancer (ICD-10: C00-C97) prior to the index date. Patients with depression (ICD-10: F32, F33), bipolar disorder (ICD-10: F30), and anxiety disorder (ICD-10: F41) diagnoses prior to or on index date were excluded. After applying similar inclusion criteria, patients without cancer were matched (1:1) to those with lymphoma based on sex, age, index year, and yearly consultation frequency. The reasons for the matching criteria were as follows: higher age and female sex are known to be associated with depression and could therefore bias the results. The index year is correlating with follow-up time and was consequently included into the matching process to enable similar follow-up durations for both cohorts. Finally, we matched for consultation frequency, as patients with cancer usually have a much higher consultation frequency than patients without cancer and this may lead to a higher likelihood of coding a diagnosis (in our case depression/anxiety). For individuals without cancer, the index date corresponded to a randomly selected visit date between January 2000 and December 2018 (Figure 1).

### Ethics

This study was conducted according to the ethical guidelines of the 1964 Declaration of Helsinki (amended, 2013). We used anonymous electronic medical records for research purposes with no directly identifiable data. Accordingly, this study did not collect informed consent from individual patients and according to German regulations, no ethical approval is needed. Anonymized data were analyzed as aggregates with no protected health information available.

### Study outcome and statistical analyses

The study outcome was the cumulative incidence of depression and anxiety disorders as function of lymphoma. After 1:1 matching, the age, sex, and yearly consultation frequency of lymphoma patients were compared with those without cancer using McNemar tests for categorical variables and paired Wilcoxon signed-rank test for continuous variables. Kaplan-Meier curves were used to compare the incidence of depression and anxiety disorders in the 10 years following the index year between the lymphoma and the non-cancer cohort. As there was no information on mortality, dead patients were considered lost to follow-up. Finally, the relationship between lymphoma, separately Hodgkin and non-Hodgkin, and both depression and anxiety disorders in the overall sample and in sex and age subgroups was investigated using Cox regression models. There were five age groups (≤40, 41–50, 51–60, 61–70, and >70 years). The results of the Cox regression analyses are presented as hazard ratios (HRs) with 95% CIs.

Power analysis was conducted using an alpha of 0.05, a power of 0.80, and two cohorts (1:1). Based on the aforementioned assumptions, the required sample size was determined to be 944 per cohort. *P*-values <0.05 were considered statistically significant. All analyses were performed using SAS 9.4.

## Results

### Baseline characteristics

This study included 4817 patients with lymphoma (687 with HL and 4130 with NHL) and 4817 patients without cancer. After 1:1 matching, there were no differences in age, sex, and consultation frequency between lymphoma cohorts and non-cancer cohorts. The average age of HL patients was 49.8 (SD: 18.2) years, and 45.6% were women. Patients with NHL were 61.9 (SD: 16.7) years in average, and 44.0% were women (Table 1).

**Table 1:** Baseline characteristics of study patients after 1:1 matching

Variable	Patients with Hodgkin lymphoma (*N*=687)	Non-cancer cohort (*N*=687)	*P* value
Women	45.6	45.6	1.000
Men	54.4	54.4	
Mean age in years (standard deviation)	49.8 (18.2)	49.8 (18.2)	1.000
Age ≤50 years	51.1	51.1	1.000
Age 51–60 years	15.7	15.7	
Age 61–70 years	18.1	18.1	
Age >70 years	15.1	15.1	
Mean number of consultations per year (standard deviation)	7.8 (8.5)	7.8 (8.5)	1.000
Variable	Patients with non-Hodgkin lymphoma (*N*=4130)	Non-cancer cohort (*N*=4130)	*P* value
Women	44.0	44.0	1.000
Men	56.0	56.0	
Mean age in years (standard deviation)	61.0 (16.7)	61.0 (16.7)	1.000
Age ≤50 years	24.1	24.1	1.000
Age 51–60 years	18.7	18.7	
Age 61–70 years	23.1	23.1	
Age >70 years	34.1	34.1	
Mean number of consultations per year (standard deviation)	9.8 (10.3)	9.8 (10.3)	1.000

Data are percentages unless otherwise specified

### Incidence of depression and anxiety in Hodgkin lymphoma patients

**Fig. 2:**
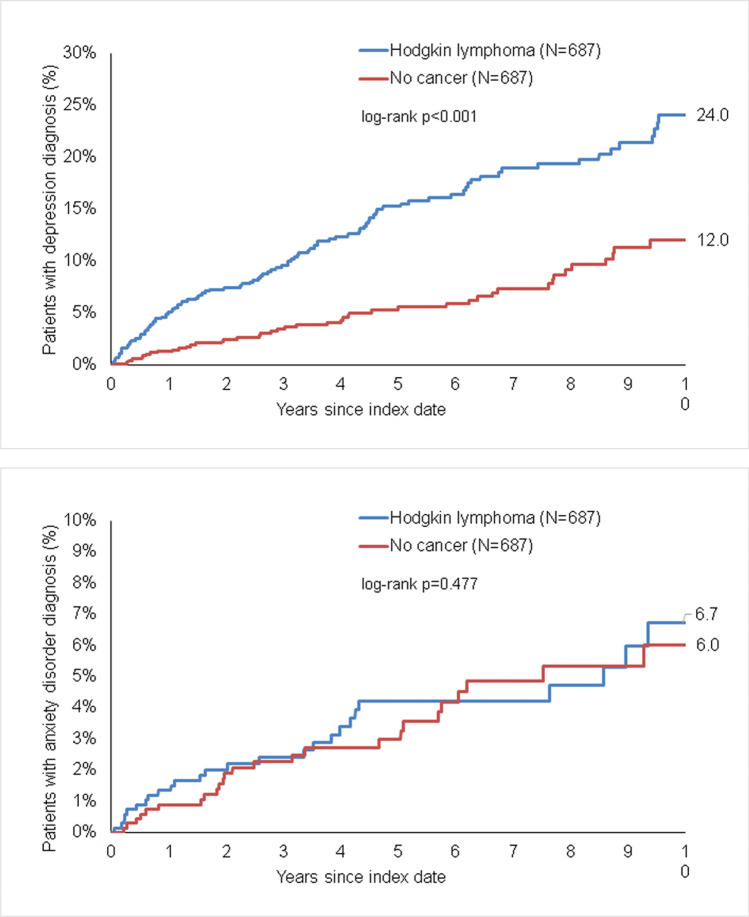
Kaplan-Meier curves for time to diagnosis of depression (upper panel) and anxiety disorders (lower panel) in patients with Hodgkin lymphoma and non-cancer patients.

The cumulative incidence of depression was significantly higher in individuals with HL than in those without cancer (24.0% versus 12.0%, *p* < 0.001) but no differences were observed in terms of anxiety disorders (6.0% vs. 6.7%, *p* = 0.477; Figure 2). The results of the Cox regression analyses are displayed in Table 2. HL was positively associated with the incidence of depression (HR 2.30, 95% CI 1.65–3.21) but not with the incidence of anxiety disorders (HR 1.21, 95% CI 0.71–2.07). The association between HL and depression was found to be significant in both women and men, as well as across different age groups, though there was only a trend for an association between HL and the incidence of depression that did not reach significance (*p* = 0.085).

**Table 2:** Association between lymphomas and the incidence of depression and anxiety disorders

	Hodgkin lymphoma		Non-Hodgkin lymphoma	
	Depression HR (95% CI)	Anxiety disorders HR (95% CI)	Depression HR (95% CI)	Anxiety disorders HR (95% CI)
Overall	2.30 (1.65–3.21) *p*<0.001	1.21 (0.71–2.07) *p*=0.478	2.09 (1.81–2.41) *p*<0.001	1.64 (1.24–2.16) <0.001
Women	1.93 (1.21.3.08) *p*=0.006	1.36 (0.62–3.00) *p*=0.443	2.02 (1.67–2.44) *p*<0.001	1.22 (0.84–1.79) *p*=0.295
Men	2.69 (1.68–4.31) *p* <0.001	1.11 (0.54–2.30) *p*=0.782	2.20 (1.67–2.44) *p*<0.001	2.29 (1.50–3.47) *p*<0.001
Age ≤50 years	2.15 (1.37–3.39) *p*<0.001	1.25 (0.59–2.62) *p*=0.561	2.44 (1.83–3.20) *p*<0.001	1.53 (0.91–2.60) *p*=0.111
Age 51–60 years	2.32 (0.89–6.05) *p*=0.085	1.03 (0.36–2.93) *p*=0.959	2.00 ((1.47–2.71) *p*<0.001	1.91 (0.95–3.86) *p*=0.071
Age 61–70 years	2.76 (1.22–6.24) *p*=0.015	1.29 (0.29–5.80) *p*=0.740	2.00 (1.47–2.73) *p*<0.001	1.58 (0.90–2.76) *p*=0.114
Age >70 years	2.43 (1.11–5.32) *p*=0.026	1.71 (0.29–10.25) *p*=0.557	1.97 (1.53–2.53) *p*<0.001	1.64 (1.01–2.68) *p*=0.047

*HR*, hazard ratio; *95% CI*, 95% confidence interval

### Incidence of depression and anxiety in non-Hodgkin lymphoma patients

**Fig. 3:**
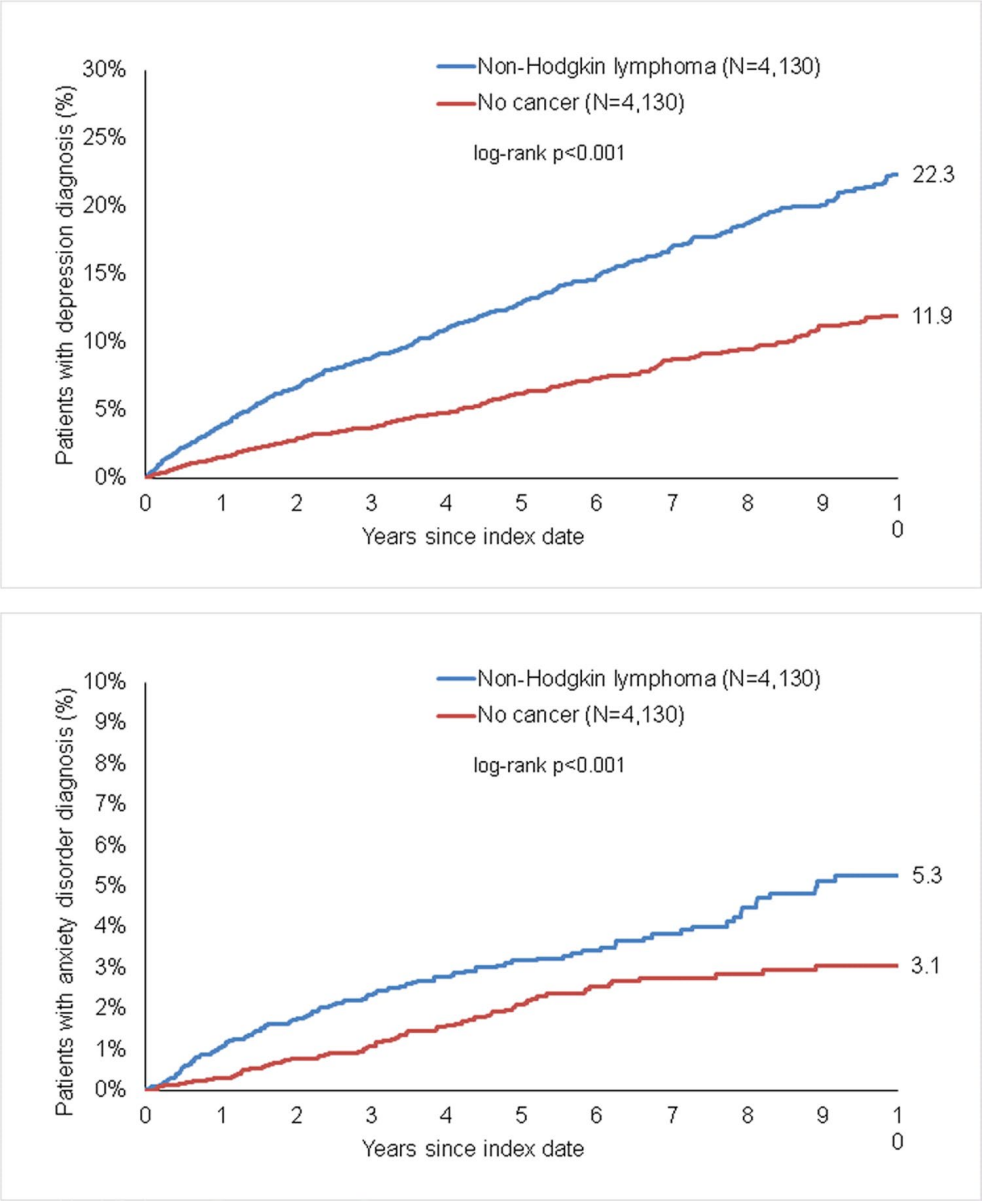
Kaplan-Meier curves for time to diagnosis of depression (upper panel) and anxiety disorders (lower panel) in patients with non-Hodgkin lymphoma and non-cancer patients.

In patients with NHL, the cumulative incidence of both depression (22.3% versus 11.9%, *p* < 0.001) and anxiety disorders (5.3% versus 3.1%, *p* < 0.001) was significantly higher than in those without cancer (Figure 3).

In regression analyses, NHL was positively associated with depression (HR 2.09, 95% CI 1.81–2.41) and anxiety disorders (HR 1.64, 95% CI 1.24–2.16) (Table 2). In subgroup analyses, the association between NHL and depression remained significant in both women and men, as well as all age groups. The association between NHL and anxiety disorders remained significant in men only. In age-stratified analyses, hazard ratios were high but did only reach significance in patients with an age >70 years (HR 1.64, 95% CI 1.01–2.68, *p* = 0.047).

## Discussion

In this study, we observed in a large cohort of patients with malignant lymphoma that the coded incidence of depression was high. Additionally, we found that HL as well as NHL was positively associated with incident depression when compared to matched individuals without cancer. We also observed an association between NHL and the incidence of anxiety disorders. The association of depression and lymphoma was equally observed in men and women as well as across different age groups.

Since patients with HL are mostly diagnosed at a young age and become long-term survivors in more than 90% with current therapy options, it is of pivotal importance to characterize the risk for the development of depression and anxiety disorders. In the current analysis comprising 687 patients with HL, the cumulative incidence of depression was significantly higher than in those without a cancer diagnosis (24.0% versus 12.0%, *p* < 0.001). This translates into a more than doubled risk increase with a HR of 2.30, which remained significant across different age groups. Interestingly, the association of incident depression was stronger in men than in women. However, we did not detect an impact of HL on the incidence of anxiety disorders. Our findings regarding a relevant association between HL and depression are comparable to a recent Danish nationwide cohort study of 945 HL patients, investigating depression and anxiety disorders by using psychotropic drug prescriptions as a proxy for these diseases. Here, the authors demonstrated that HL patients had higher 5‐year cumulative incidence of receiving a prescription for a psychotropic drug (21.5%) as compared to a matched cohort (8.4%) [6].

Besides the detrimental effect of HL on depression and anxiety disorders, we also investigated the impact of NHL on these mental diseases. In our cohort of 4130 NHL patients with a mean age of 61.9 years, the cumulative incidence of both depression (22.3% versus 11.9%, *p* < 0.001) and anxiety disorders (5.3% versus 3.1%, *p* < 0.001) was significantly higher than in those without cancer. Interestingly, the association between NHL and the incidence of depression remained robust across all age groups and in both men and women. These findings are in line with a smaller French study investigating the incidence of psychotropic drug use during the diagnosis and active treatment phase in patients with B-NHL. Here, among 745 newly diagnosed B-NHL patients with a mean age of 65.1 years, psychotropic treatment—reflecting the possible prevalence of depression or anxiety disorders—was initiated in 31.5 %, compared to 7.6% in the general population during the same period [18]. Another study conducted by Oerlemans et al. analyzed data of 489 patients with HL or DLBCL who serially completed the Hamilton Anxiety and Depression Scale [19]. Here, anxiety and depressive symptoms were more frequently reported by lymphoma patients compared to a normative population.

The current study expands the existing literature by analyzing a very large dataset of patients and analyzing the coded incidence of depression and anxiety disorders instead of medication prescriptions or questionnaire results. The results presented in this study support the hypothesis that lymphoma has a relevant influence on the occurrence of depression and NHL is additionally associated with the occurrence of anxiety disorders. Additionally, we are able to give a robust estimate on the risk increase in these patients.

The prevalence rates of mental disorders may be notably higher during the episode of cancer diagnosis and following chemotherapy, due to the high degree of perceived uncertainty and fear. Still, impaired mental health often remains unnoticed by health care providers [17, 20]. Singer et al. investigated the frequency of mental health conditions in cancer patients that are treated in acute care hospitals in a meta-analysis of eight studies, indicating prevalence rates ranging from 23 to 53% [21]. Although, the studies in this meta-analysis were performed with inpatient with heterogenous cancer diagnosis, these and our current analysis strongly support the notion that comprehensive cancer care should also include testing for psychological distress. Oncologists in acute care hospitals, as well as general practitioners, have to be aware that their cancer patients are at higher risk for depression or anxiety and therefore the implementation of screening instruments during consultations should be recommended. Here, valuable tools like the Patient Health Questionnaires (PHQ2, PHQ9) [16], the Hamilton Anxiety and Depression Scale, or the NCCN Distress Thermometer [22] may serve as an initial step in the assessment of mood disorders in the primary care setting [23].

Since therapeutic options for both HL and NHL have improved over the past decades, many patients become cancer survivors. Multimodal therapy consisting of chemotherapy, immune therapy, or radiation can lead to complete remission, but may result in a substantial amount of long-term sequelae apart from depression, such as chronic fatigue, neuropathy, neurocognitive decline, sexual changes, and many more [24]. Our study demonstrates that lymphoma patients are not only at increased risk for depression and anxiety disorders during the initial treatment phase but also during long-time follow-up. Here, patients are regularly treated by their respective primary care practitioners. Our data highlight that, related to the high incidence of depression in patients with lymphoma during long-time follow-up, screening could be warranted not only by specialists but also primary care practitioners. This is of pivotal importance because there is evidence that combined pharmacological and psychological treatment for depression is effective in improving levels of depression and quality of life for cancer patients, though not survival [25]. Additionally, a meta-analytic review suggested that psychological interventions may assist some cancer patients in reducing levels of anxiety [26].

The following limitations need to be acknowledged. First, this study has the weaknesses inherent to all database analysis, as it relies on ICD-10 codes for establishing diagnoses. Thus, the extent of underlying misclassification related to miscoding or undercoding of diagnoses cannot be assessed. However, the German Disease Analyzer database has been used, and its reliability has been validated in several medical studies [14, 15, 27]. Second, the Disease Analyzer database does not capture detailed laboratory values nor information on disease stages or cancer relapse of patients with HL or NHL. Therefore, our study lacks information regarding disease severity, current treatment, and, importantly, remission status and we are unable to account for these. Additionally, our study lacks more granular information on, e.g., education and employment status. These may have impact on the incidence of depression and anxiety in our cohorts that we are unable to adjust for. Last, due to a limited differentiation in coding and the sample size, we are unable to conduct subgroup analyses of the different NHL types and investigate their impact on the incidence of depression or anxiety disorders. This is a confounder of our study, since NHL subtypes have different outcomes in terms of long-term survival and overall survival depending on stage and prognostic factors and therefore the incidence of depression and anxiety disorders may differ among NHL subtypes depending on the individual prognosis. Nevertheless, we were able to demonstrate a robust association between NHL per se and depression or anxiety disorders.

In conclusion, this study demonstrates that the coded incidence of depression is high in German patients with lymphoma. This resulted in a robust association between HL as well as NHL with an increased incidence of depression compared to matched non-cancer individuals. Additionally, we observed an association between NHL and the incidence of anxiety disorders. Therefore, patients with malignant lymphomas should be closely screened for depression and anxiety disorders even several years after the initial diagnosis of the disease.

## Data Availability

The data that support the findings of this study are available from KK and IQVIA. Restrictions apply to the availability of these data, which were used under license for this study. Data are available from KK with the permission of IQVIA.

## Abbreviations

CI: Confidence interval
FL: Follicular lymphoma
HL: Hodgkin lymphoma
HR: Hazard ratio
ICD: International Classification of Diseases
NHL: Non-Hodgkin lymphoma
